# Lu_2_O_3_:Pr,Hf Storage Phosphor: Compositional and Technological Issues

**DOI:** 10.3390/ma7010157

**Published:** 2013-12-31

**Authors:** Aneta Wiatrowska, Eugeniusz Zych

**Affiliations:** Faculty of Chemistry, University of Wroclaw, 14 F. Joliot-Curie Street, Wroclaw 50-383, Poland; E-Mail: aneta.wiatrowska@philips.com

**Keywords:** Lu_2_O_3_:Pr,Hf ceramics, storage phosphors, thermoluminescence, energy traps

## Abstract

Lu_2_O_3_:Pr,Hf ceramics were investigated using mainly thermoluminescence (TL) technique. Their ability to efficiently store energy acquired upon irradiation with X-rays was proven. The best TL performance was achieved for compositions containing 0.025%–0.05% of Pr and about 0.1% of Hf. Further enhancement of TL efficiency was attained by increasing the temperature of sintering of the ceramics up to 1700 °C and applying reducing atmosphere of forming gas. It was also proven that fast cooling after the sintering at 1700 °C significantly enhanced the storage phosphor performance. TL glow curve contained three components peaking around 130, 250 and 350 °C. Among them, the one at 250 °C contributed the most to the total TL.

## 1. Introduction

Persistent and storage phosphors form a specific class of luminescent materials. In such compositions the excited electron after absorption of the incident photon gives no immediate emission but is immobilized in an excited state for quite a long time. Once irradiated, persistent phosphors continue releasing the acquired energy at room temperature for minutes or hours afterwards, while storage phosphors rather retain it for days or even years if no external stimulation—thermal or optical—is applied [1–3]. The difference comes from the depth of energy-traps present in the materials. Yet, both groups of phosphors share the mechanisms of storing and subsequent releasing of the gained energy.

A significant number of new, efficient persistent phosphors have been discovered in last two decades as a consequence of Matsuzawa’s work on SrAl_2_O_4_:Eu^2+^,Dy^3+^ [4,5], and SrAl_2_O_4_:Eu^2+^,Nd^3+^ [5] showing an extraordinary performance. Soon, new efficient persistent phosphors were reported: Sr_3_SiO_5_:Eu^2+^,Nd^3+^ [6,7], Ba_4_(Si_3_O_8_)_2_:Eu^2+^,Dy^3+^ [8], Sr_3_Al_2_O_5_Cl_2_:Eu^2+^,Tm^3+^ [9], CaAl_2_O_4_:Eu,Nd [4], Sr_2_MgSi_2_O_7_:Eu,Dy [10]; Sr_4_Al_14_O_25_:Eu,Dy [11]; Ca_2_Si_5_N_8_:Eu,Tm [12]; YPO_4_:Ce^3+^,Ln^3+^ (Ln = Nd, Er, Ho, Dy) [13–15], YPO_4_:Pr^3+^,Ln^3+^ (Ln = Nd, Er, Ho, Dy) [16]. This research allowed getting much better insight on physics of such materials. These findings almost coincided in time with presentation of a method of positioning of lanthanides’ levels against valence and conduction bands in various hosts by Dorenbos [17,18]. His approach appeared very useful in more deliberate prediction of possible energy storing by particular dopants in specific lattices.

Research in the area of storage phosphors did not bring so spectacular results if new compositions are considered. Still, the mostly used storage phosphors are, discovered years ago, (Ba,Sr)FBr_1−_*_x_*I*_x_*:Eu^2+^ [19,20] and CsBr:Eu^2+^ [21], CaSO_4_:Dy [22], LiF:Mg,Cu,P [22,23] and some others the reader may find in reviews [1–3]. (Ba,Sr)FBr_1−_*_x_*I*_x_*:Eu^2+^ and CsBr:Eu^2+^ are proven to fulfill the requirements of digital radiography, though some of their parameters are rather far from “ideal” values. For example, both have quite low densities (BaFBr: 4.96 g/cm^3^, CsBr: 4.44 g/cm^3^), which reduces their absorption coefficients for X-rays, especially the more energetic. Detailed comparisons of that parameter for different compositions can be easily made using the XCOM service delivered by NIST [24]. Thus, LiF and CaSO_4_ (2.64 g/cm^3^ and 2.96 g/cm^3^, respectively) have almost constant, and rather low absorption coefficients at higher energies, while the more dense materials, containing elements of higher *Z* numbers, show much higher, strongly increasing for lower energies, effectiveness to absorb γ- or X-rays. Consequently, this difference divides the storage phosphors into two groups: those of low densities, which are useful in personal dosimetry, and the high-density compositions that are more appropriated for imaging and whenever enhancement of probability of absorbing the incoming photons matters. [25]. Yet, other applications are also possible [26]. Among the storage phosphors, those containing Lu_2_O_3_ as the host are exceptional. The reason is the high effective Z number of lutetium oxide (67.3) and its very high density (9.42 g/cm^3^), which situate Lu_2_O_3_-based phosphors among the most efficient absorbers of γ- and X-rays.

We have already published some data on energy storing in Lu_2_O_3_-based compositions [27–29]. In the present paper we deal with the influence of some technological and compositional issues—temperature and atmosphere of fabrication, concentration of the activators, cooling rate and the procedure of preparing the starting powders on the capability of energy storing by Lu_2_O_3_:Pr,Hf sintered ceramics.

## 2. Results and Discussion

### 2.1. Thermoluminescence

**Figure 1.**
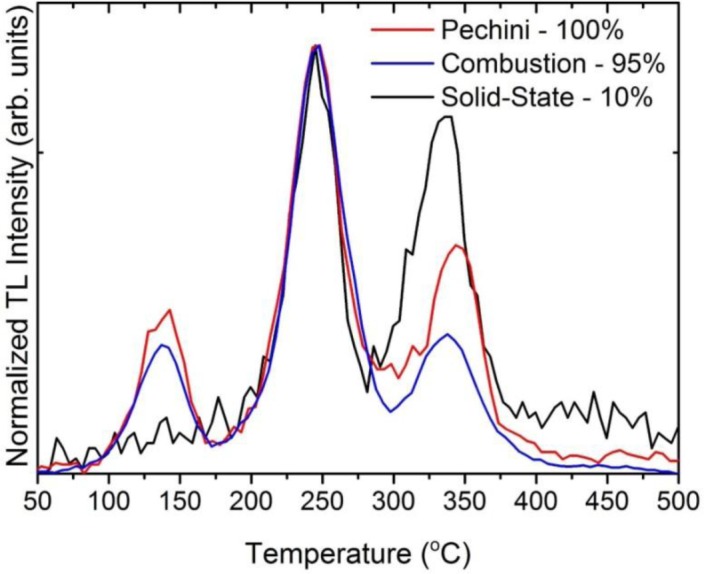
Thermoluminescence (TL) glow curves of Lu_2_O_3_:0.05%Pr,0.1%Hf ceramics sintered at 1700 °C in 25%H_2_+75%N_2_ using different starting powders—prepared by Pechini or combustion method or mixture of commercial oxides. The accuracy of the temperature for all TL experiments is ±2.5 °C.

We shall see later that efficient thermoluminescence (TL) requires preparation of the ceramics at temperature as high as 1700 °C (the highest temperature we can routinely apply in our furnaces). Therefore let us start comparing the TL glow curves of ceramics prepared at such a temperature and N_2_-H_2_ mixture using initial powders prepared in different manners: the Pechini technique, combustion synthesis or by mixing commercial oxides. The thermoluminescence (TL) glow curves of three ceramics made using such powders are given in Figure 1.

It is immediately seen that the TL efficiency of the materials made using the commercial oxides is by almost an order of magnitude lower than from the other two specimens. This effect was repetitively observed. The other two ceramics performed almost identically. We concluded that the powders for sintering should be prepared through procedure allowing for atomic mixing, as both in combustion and in Pechini synthesis the reactants are first dissolved in water, and then processed towards nanocrystalline Lu_2_O_3_:Pr,Hf powders. This, we think, allows for much better uniformity of the starting powders even if the thermally stimulated diffusion of the material constituents is limited in the refractory oxide. Getting such an uniformity of the final material starting with commercial oxides would require diffusion of the various components over significant distances in the solid phase during its sintering. Evidently, from the data given in Figure 1, we may conclude that such an efficient homogeneous distribution of the dopants is not achievable through all-solid-phase procedure. This effect is not unusual. The broadly used Y_2_O_3_:Eu red emitting powder phosphor should be prepared by co-precipitation and subsequent thermal decomposition to achieve high performance. Solid state reaction reduced its efficacy by a factor of 2–3 [30]. Consequently, all the other experimental results we shall present for ceramics made of powders prepared with Pechini method. We prefer it over the combustion procedure as it allows larger quantities to be made and, being less vigorous, is also easier to control.

**Figure 2.**
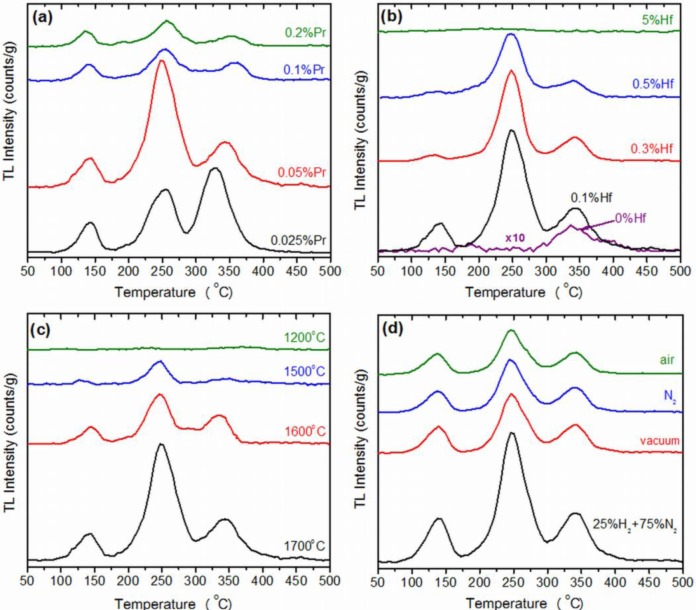
(**a**) TL glow curves of Lu_2_O_3_:*x*%Pr,0.1%Hf (*x* = 0.025%, 0.05%, 0.1%, 0.2%) ceramics annealed at 1700 °C in 25%H_2_ + 75%N_2_; (**b**) TL glow curves of Lu_2_O_3_:0.05%Pr,*x*%Hf (*x* = 0%, 0.1%, 0.3%, 0.5%, 5%) ceramics annealed at 1700 °C in 25%H_2_ + 75%N_2_. Note that the signal for the Pr singly doped samples was multiplied by 10 to make it visible; (**c**) TL glow curves of Lu_2_O_3_:0.05%Pr,0.1%Hf ceramics annealed at different temperatures in 25%H_2_ + 75% N_2_; (**d**) TL glow curves of Lu_2_O_3_:0.05%Pr,0.1%Hf ceramics annealed at 1700 °C in different atmospheres.

Figure 2a,b present the TL glow curves recorded for Lu_2_O_3_:Pr,Hf ceramics made at 1700 °C in reducing atmosphere and containing Pr and Hf at different concentrations. In Figure 2a the Hf concentration is 0.1 wt%. Lu and only the Pr percentage varies in the range of 0.025%–0.2%. In Figure 2b this is the concentration of Pr which is fixed at 0.05% and Hf content ranges between 0% and 5%. The general view is that TL intensity is extremely sensitive to Pr content. The total TL intensity is the highest and very similar for two compositions: Lu_2_O_3_:0.05%Pr,0.1%Hf and Lu_2_O_3_:0.025%Pr,0.1%Hf. Already for 0.1% of Pr the TL intensity gets reduced by a factor of about 4 and becomes even less efficient when the Pr concentration is yet doubled. Clearly, the efficient TL requires low Pr concentrations. We should note, however, that the TL glow curves of the two best samples—Lu_2_O_3_:0.05%Pr,0.1%Hf and Lu_2_O_3_:0.025%Pr,0.1%Hf—differ significantly especially in the high temperature range. When the Pr content is 0.05% the component around 250 °C dominates the glow curve, while for yet lower concentration of the activator, 0.025%, the most efficient TL appears around 330 °C. Still, for this composition the band around 250 °C comprises noticeable fraction of the total TL intensity. Apart from the high-temperature TL components, all samples give some thermoluminescence around 130 °C. Traps of such characteristics are able to free carriers even at room temperature [31]. Indeed, we found that TL glow curves recorded a few hours after the samples irradiation were perfectly flat up to 170 °C proving that 4 h of delay was enough to empty the trap(s) responsible for the TL at 130 °C. Details of this issue were discussed in [28].

Figure 2b presents TL glow curves for samples with fixed Pr concentration of 0.05% and varying content of Hf. The shape of the curves is very similar for all concentrations. Yet, the TL intensity reaches the highest level when the Hf content is 0.1%. Above this value the TL efficiency gets reduced continuously though not rapidly. Hence, the ability for energy storage in Lu_2_O_3_:Pr,Hf is tremendously susceptible to the concentration of Pr and less, though noticeably, sensitive to the content of Hf. Yet, both dopants are needed to form an efficient energy-trapping system in the lutetia host. In the next step, we shall try to determine the optimal sintering temperature and atmosphere of the Lu_2_O_3_:Pr,Hf storage phosphors.

Figure 2c,d gives general information on the influence of two parameters, sintering temperature and atmosphere, on the final products’ TL intensity. Independently on the atmosphere of preparation the ceramics give significant TL signals and the shapes of the glow curves remain similar. Also the temperature of sintering does not change the shape of the glow curves to any significant degree. However, intensity of thermoluminescence is strongly dependent on both sintering temperature and its atmosphere. With increasing temperature of preparation the TL intensity got continuously enhanced and between 1500 and 1700 °C the increase was truly severe, at least by a factor of five (Figure 2c). As seen in Figure 2d, the most significant TL signal was consistently recorded for specimens fabricated at reducing atmosphere of N_2_-H_2_ mixture. Nevertheless, neither of the applied atmospheres totally annihilated the TL. This is the *effectiveness* of storing energy in the material, which was influenced by the atmosphere of fabrication.

**Figure 3.**
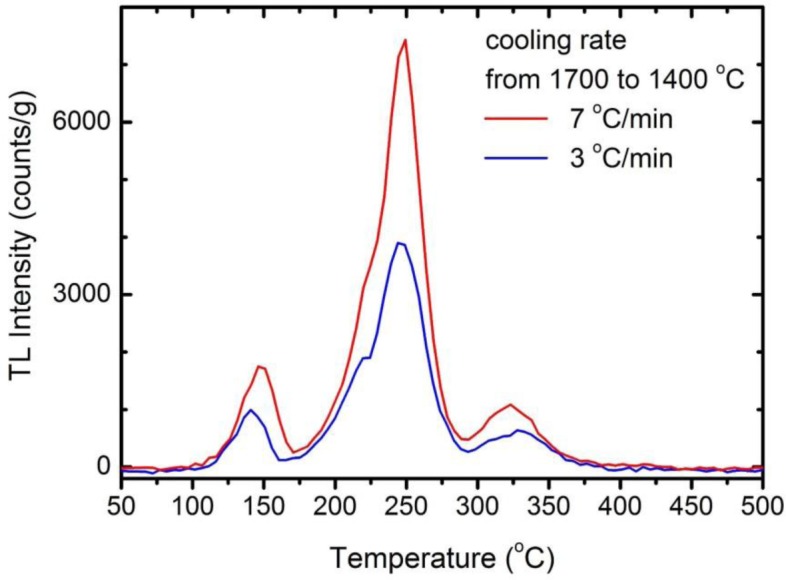
TL glow curves of Lu_2_O_3_:0.05%Pr,0.1%Hf ceramics annealed for 5 h at 1700 °C in forming gas. One sample was cooled at the rate of 7 °C/min in the range of 1700–1400 °C and 3 °C/min below 1400 °C, while the other one at the constant rate of 3 °C/min.

Finally, we decided to check if the cooling rate after sintering at 1700 °C may play a role. We prepared two ceramics: Lu_2_O_3_:0.05%Pr,0.1%Hf annealed for 5 h at 1700 °C in a forming gas with different cooling rates. From the same batch of raw powder we made a ceramic with the regular cooling of 3 °C/min and another one applying the cooling rate of 7 °C/min in the most crucial range of 1700–1400 °C, while below 1400 °C the rate was again 3 °C/min. Comparison of the two TL glow curves is given in Figure 3. Clearly, the quickly cooled specimen showed TL intensity enhanced by a factor of almost 2. It is noteworthy that all three TL bands became more potent to the same degree, roughly. One may note, that the position of the TL peaks changed slightly too but we did not track this effect any further. Hence, we see that not only the high-temperature of fabrication is beneficial, but also that faster cooling preserves the high-temperature equilibrium and more energy-trapping sites outlast down to room temperature. Thus, it appears that, during the cooling, a fraction of the traps formed at the high temperature of the phosphors preparation may be extinguished if the process is slow. It raises the question about possible reasons of that effect and we shall deal with that later.

#### 2.1.1. Evolution of Morphology during Heat-Treatment

**Figure 4.**
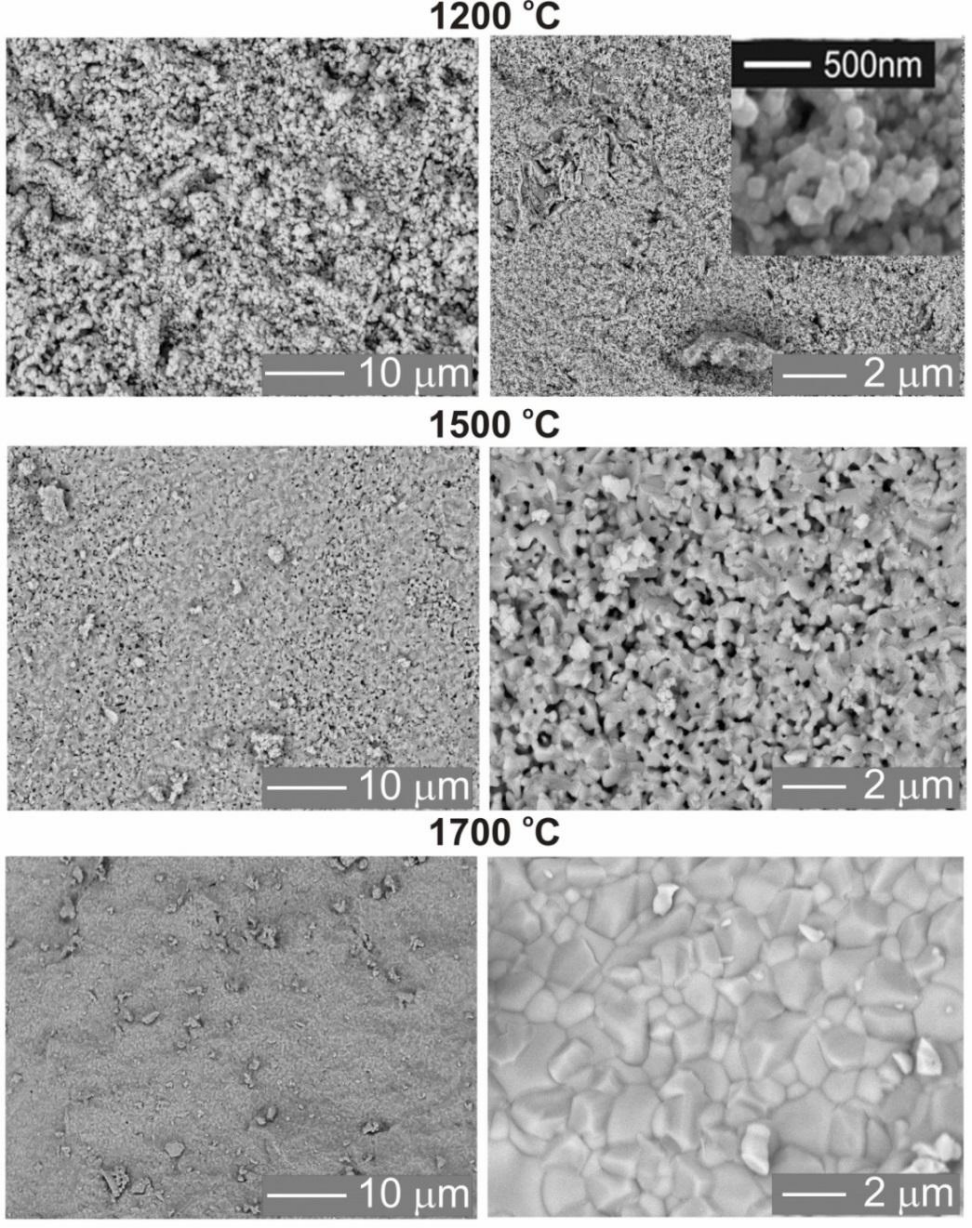
SEM images of Lu_2_O_3_:0.05%Pr,0.1%Hf ceramics annealed in a mixture 25%H_2_ + 75%N_2_ for 5 h at different sintering temperatures 1200 °C, 1500 °C and 1700 °C.

Figure 4 shows SEM images of Lu_2_O_3_:0.05%Pr,0.1%Hf ceramics obtained at various sintering temperatures: 1200 °C (Figure 4a), 1500 °C (Figure 4b) and 1700 °C (Figure 4c). Evolution of the morphology with sintering temperature appears to mirror growth of energy storing efficacy presented in Figure 2c and discussed above.

The sintering temperature has a substantial influence on the average grains sizes, their packing and the whole body porosity. Ceramics obtained at the 1200 °C are very porous, the grains are spherical with very similar diameters of about 200 nm (Figure 4a). This confirms that at 1200 °C only a very limited mass transfer takes place. Increasing the sintering temperature up to 1500 °C (Figure 4b) leads to an enlargement of grains to an average size of about 400–800 nm, development of a network of connections between them, and reduced porosity which, however, is still significant. Confirmation of these observations is also found in the XRD patterns (Figure 6), where the diffraction lines of the ceramics made at 1200 °C are clearly broader compared to those of the specimens made at 1500 °C and 1700 °C. This is in agreement with the Scherrer’s equation, according to which the width of diffraction line is inversely proportional to the crystallite size [32,33]. Finally, the ceramics sintered at 1700 °C have a much different morphology (Figure 4c) indicating a substantial rearrangement and relocation of the material building entities. The grains are much larger, mostly 2–5 μm, have quite regular shapes but with clearly developed plain surfaces and presence of classic triple points where corners of grains meet. The porosity is practically removed and the grain boundaries are well defined and narrow. Clearly, sintering at 1700 °C is connected with a very severe mass transfer, during which some grains grow at the expense of others, which disappear. The grains are then re-formed practically from scratch at the high temperature of sintering, and their volume enlarges by almost three orders of magnitude.

**Figure 5.**
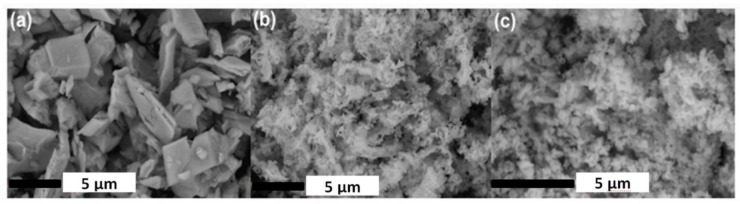
SEM images of the powders used to make sintered ceramics. (**a**) Mixture of the commercial oxides; (**b**) powder made by means of combustion with glycine; and **(c)** powder prepared with classic Pechini technique. Note the large grains characteristic for the mixed commercial powders, which greatly hinders their sintering (see Figure 7).

**Figure 6.**
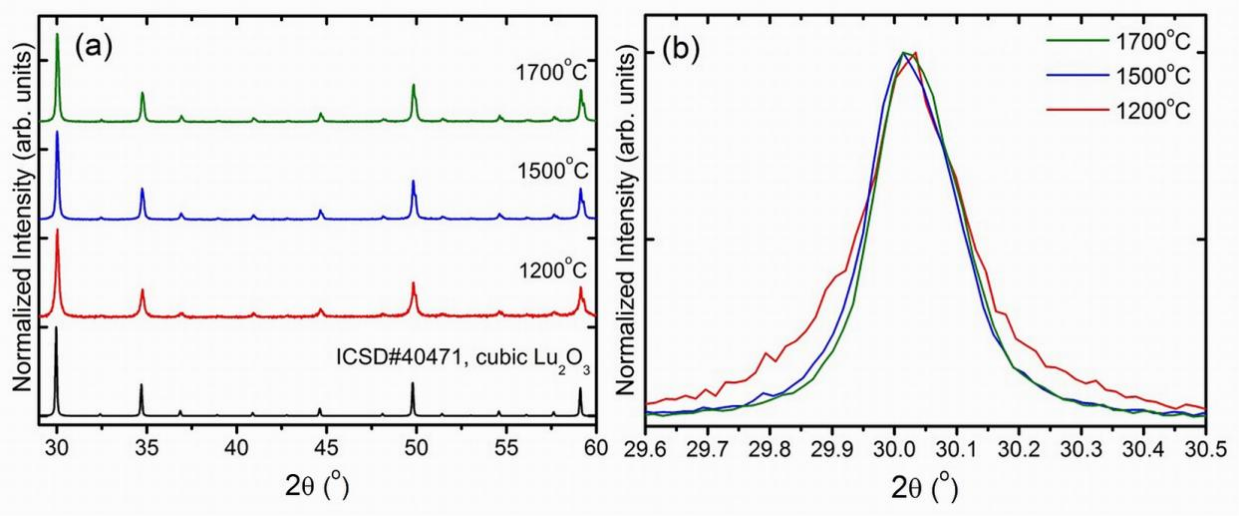
(**a**) XRD patterns of Lu_2_O_3_:0.05%Pr,0.1%Hf ceramics annealed in mixture of 25%H_2_ + 75%N_2_ for 5 h at different sintering temperatures: 1200 °C, 1500 °C and 1700 °C in the range of 29–60 degree; (**b**) Broadening of the line around 29.6–30.5 degree proves that after heating at 1200 °C the crystallites are noticeably smaller.

**Figure 7.**
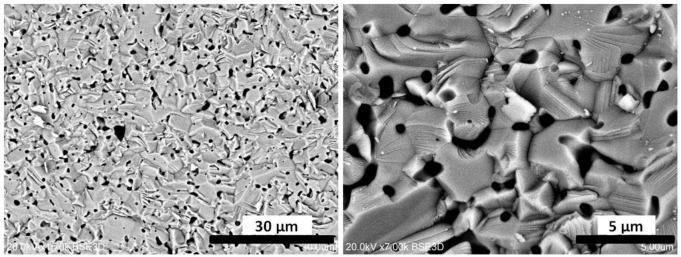
SEM images of the ceramic specimen prepared by sintering the mixture of commercial oxides, Lu_2_O_3_, Pr_4_O_11_ and HfO_2_, at 1700 °C. Comparison with the image of the starting powders (Figure 5a) confirms that the interdiffusion of the components is only superficial, giving insubstantial distribution of the activators throughout the ceramic body.

Details of the physics and mechanism of energy storing and thermoluminescence in Lu_2_O_3_:Pr,Hf were already given in [28] and we shall not repeat the considerations here. Let us yet note that the most significant change of morphology occurs between 1500 and 1700 °C, which mirrors the range of temperatures within which the most substantial enhancement of TL efficacy occurs. We conclude that this is the high-temperature transformation of the matter towards a body with large grains, when the population of energy trapping centers able to generate efficient thermoluminescence is greatly enhanced. Pretty similar change of morphology was observed during sintering of the combustion-made powder. Therefore we do not present the images here. It is not surprising comparing the morphology of the starting powders, given in Figure 5. This figure also reveals the radically different morphology of the mixture of commercial oxides used for sintering of a reference sample showing a very inefficient TL (see Figure 1). The mixed oxides are composed of particles a few microns in diameter. Hence, these particles are by about 2 orders of magnitude in diameter larger than those obtained from Pechini or combustion synthesis. In fact, the grains in the commercial powders are at least as large as the grains in ceramics obtained by sintering at 1700 °C of either of the two other powders, made by means of Pechini or combustion method (Figure 4). Thus, it is not surprising that the commercial powders do not sinter efficiently, and the mass transfer allowing for efficient diffusion of the starting mixture components is far too inadequate to give a uniform distribution of the components and consequently the efficient TL. This is fully confirmed by SEM images of the ceramic specimen made by sintering the mixture of commercial oxides presented in Figure 7. Abundant voids are present throughout the body, the grains are irregular in shape, and their sizes are not much different from what was seen before sintering (Figure 5a). Clearly, the commercial powders, even at 1700 °C, do not sinter efficiently and thus the interdiffusion of the three oxides, Lu_2_O_3_, Pr_4_O_11_ and HfO_2_, is far too deficient to attain their optimized distribution throughout the whole body of the phosphor pellet.

Even in large single crystals, high-temperature treatment facilitates migration of constituent entities—atoms and defects. At high temperatures a formation of new defects, mostly vacancies, by expelling the atoms from surface to the vacuum takes place [33,34]. In ceramics sintered using the fine nanocrystalline starting powders a large fraction of the grains, in practice, the whole body, is re-built atom-by-atom from scratch. This was clearly depicted in Figure 4. It was discussed in literature that such conditions favor an agglomeration of defects, as this may be easily thermodynamically preferred [35,36]. Thus, it appears that during the high-temperature sintering of the nanocrystalline powders the defects introduced during preparation diffuse effectively throughout the forming ceramic body and presumably form clusters well distributed throughout the pellet. Lu_2_O_3_:Pr,Hf ceramics composed in such a way are apparently able to efficiently trap free carriers—electrons and holes. Hf(IV) is assumed to be responsible for immobilization of electrons, while at the same time, Pr(III) may trap holes due to its ability to transform towards Pr(IV). Holes may be also trapped by the interstitial oxygen due to its negative net charge. Yet, its population is limited as, even at reducing atmosphere, the Hf(IV), with its positive net charge, may hinder (though not exclude) the generation of such a defect.

## 3. Experimental Section

Samples were prepared as sintered ceramics. The starting powders of appropriate compositions were uniaxially pressed into 7 mm pellets under the load of 5 tons and were then sintered in a tube furnace at 1200, 1500, or 1700 °C in vacuum, H_2_ + N_2_ mixture (1:3 by volume), nitrogen or air atmospheres for 5 h. Sintering for 1 h gave samples with TL by a factor of 4–5 lower compared to specimens sintered for 5 h. Consequently, we present data only for materials sintered for 5 h. The typical heating and cooling rates were 3 °C/min. For comparison, one sample was prepared with the cooling rate of 7 °C/min in the range of 1700–1400 °C and 3 °C/min below 1400 °C in the reducing mixture.

Since the most powerful thermoluminescence was routinely observed for ceramics made at the highest temperature (1700 °C) and the most reducing conditions (H_2_ + N_2_ mixture) the influence of the Pr and Hf content on TL was systematically tested only for samples made at such conditions. The starting powders for sintering were typically prepared by the standard Pechini technique [37]. Yet, for comparison, also powders made by means of glycine (NH_2_CH_2_COOH) combustion technique [38] as well as using thoroughly-ground mixture of oxides, Lu_2_O_3_ (4N5), Pr_6_O_11_ (5N), HfO_2_ (3N), were sintered at 1700 °C in 25%H_2_ + 75%N_2_ mixture.

X-ray powder diffraction technique using Bruker D8 Advance diffractometer with Ni-filtered Cu K_α__1_ radiation (λ = 1.5418 Å) was applied to examine the samples phase purity. The XRD patterns were recorded in the range of 2θ = 29–60 degree with the step of 2θ = 0.00857 degree and the counting time of 0.2 s per point. Micrographs were recorded with the use of a Hitachi S-3400N Scanning Electron Microscope (SEM) equipped with an energy dispersive X-ray spectroscopy (EDAX) analyzer. For the experiments freshly broken pellets of sintered ceramics were sputter coated with an ultrathin layer of gold.

Thermoluminescence (TL) glow curves were recorded in the range of 20–500 °C using a custom-made temperature controller with linear heating rate and using Ocean Optics HR2000 CG spectrometer operating under the control of a Spectra Suit dedicated software. The thermoluminescent photons were collected with a 74-UV lens coupled to a QP600-1-SR waveguide. TL glow curves were recorded with the counting time of 1 s and monitoring the intensity of the Pr^3+^ red emission band around 630 nm. Since the spectrometer used CCD to record light, full spectrum in the range of 200–1100 nm was also taken every 1 s during heating. These spectra proved that only the red luminescence of Pr around 590–690 nm was produced. The typical heating rate was 4.8 °C/s and accuracy of the temperature is estimated to be ±2.5 °C. White X-Rays from a Cu X-ray tube were used for irradiation of the materials prior to thermoluminescence experiments.

## 4. Conclusions

In this paper we presented results showing that Lu_2_O_3_:Pr,Hf ceramics were able to store energy when irradiated with X-rays. Efficiency of this process, as monitored by thermoluminescence intensity, was showed to strongly depend both on Pr and—to a slightly lesser degree—on Hf concentration. The optimal content of the dopants were found to be 0.025%–0.05% (Pr) and about 0.1% (Hf). It was also proven that high temperature of preparation (1700 °C) and reducing atmosphere of the forming gas were beneficial for the storage efficacy. Yet, each of the applied preparation atmospheres—forming gas, vacuum, nitrogen, air—allowed for energy storing in Lu_2_O_3_:Pr,Hf ceramics. Good stability of the TL signal (presented in [28]) is an important property for practical uses. Since lutetia has very high density of 9.4 g/cm^3^ it assures very efficient absorption of ionizing radiation (gammas and X-rays) and thus it could be considered as a dosimetric material is some specific areas. We continue this research towards Lu_2_O_3_:Pr,Hf powder storage phosphor with low agglomeration to have some freedom in making larger screens, for example in the form of composed plates. This is a hard task, as the required high temperature of preparation naturally enhances aggregation of grains.
